# Phase I/Ib study of olaparib and carboplatin in women with triple negative breast cancer

**DOI:** 10.18632/oncotarget.16577

**Published:** 2017-03-25

**Authors:** Jung-Min Lee, John L. Hays, Victoria L. Chiou, Christina M. Annunziata, Elizabeth M. Swisher, Maria I. Harrell, Minshu Yu, Nicolas Gordon, Tristan M. Sissung, Jiuping Ji, William D. Figg, Lori Minasian, Stanley Lipkowitz, Bradford J. Wood, James Doroshow, Elise C. Kohn

**Affiliations:** ^1^ Women’s Malignancies Branch, Center for Cancer Research, National Cancer Institute, Bethesda, MD, USA; ^2^ Division of Medical Oncology, Department of Internal Medicine, The James Comprehensive Cancer Center, The Ohio State University Wexner Medical Center, Columbus, OH, USA; ^3^ Division of Gynecologic Oncology, Department of Obstetrics and Gynecology, University of Washington, Seattle, WA, USA; ^4^ Genitourinary Malignancies Branch, Center for Cancer Research, National Cancer Institute, Bethesda, MD, USA; ^5^ National Clinical Target Validation Laboratory, Leidos Biomedical Research Inc, Frederick National Laboratory for Cancer Research, Frederick, MD, USA; ^6^ Center for Interventional Oncology, Radiology, and Imaging Sciences, Clinical Center and National Cancer Institute, NIH, Bethesda, MD, USA; ^7^ Division of Cancer Treatment and Diagnosis, National Cancer Institute, NIH, Bethesda, MD, USA

**Keywords:** triple negative breast cancer, olaparib, carboplatin

## Abstract

**PURPOSE:**

To investigate the safety, activity, and potential biomarkers of response to olaparib and carboplatin combination in sporadic triple negative breast cancer (TNBC). EXPERIMENTAL DESIGN: Metastatic or recurrent TNBC patients with no germline *BRCA* mutation or with BRCAPro scores <10% and a negative family history were eligible. A 3+3 dose escalation tested olaparib capsules (400mg bid, days1-7) with carboplatin AUC3-5 on day1 or 2 every 21 days, ≤ 8 cycles, with olaparib 400mg bid maintenance. Peripheral blood mononuclear cells were collected for polymorphisms and PAR levels, and paired tumor biopsies (pre-/post-cycle 1) for proteomics and apoptosis endpoints.

**RESULTS:**

28 women were treated (median 5 prior regimens [0-12]). Dose-limiting toxicity was thrombocytopenia, and symptomatic hyponatremia with carboplatin AUC5. The maximum tolerated dose was olaparib 400mg bid+carboplatin AUC4. Grade 3 and 4 adverse events included neutropenia (36%), thrombocytopenia (11%), and anemia (11%). Responses included 1 complete response (CR; 69^+^months) and 5/27 partial responses (19%; median 4months [4-7]), for a response rate of 22%. Biomarker findings did not correlate with response. The long-term CR patient with prior negative *BRCA* testing was found to have deletion of *BRCA1* exons1-2.

**CONCLUSIONS:**

The olaparib/carboplatin combination is tolerable and has modest activity in sporadic TNBC patients. Further evaluation of predictive biomarkers to identify those with *BRCA* wild type who had response is warranted.

## INTRODUCTION

Triple-negative breast cancer (TNBC), defined by lack of expression of estrogen receptor, progesterone receptor, and *HER2* gene amplification, accounts for approximately 15% of breast cancers [1]. TNBC is a heterogeneous group of tumors with more aggressive clinical features [2]. The Cancer Genome Atlas (TCGA) analysis demonstrated that frequent loss-of-function and gain-of-function alterations in TNBC involving genes associated with the DNA damage repair and phosphatidylinositol 3-kinase (PI3K) pathways [3]. Homologous recombination repair (HRR) is a predominantly error-free DNA double-strand break repair mechanism [4]. Key components of the HRR pathway include the tumor-suppressor proteins BRCA1 and BRCA2 [4]. DNA damage repair pathways are attractive therapeutic targets in TNBC with germline or somatic HRR dysfunction [1, 2]. We previously reported experience with the polyADP ribose polymerase (PARP) inhibitor (PARPi) olaparib for women with germline *BRCA* mutation [5]. We now examine the ability to extend those findings to women with TNBC who do not have intrinsic HRR dysfunction.

Approximately 15% of TNBC harbor a germline mutation in *BRCA1* or *BRCA2*; up to 80% of *BRCA1* mutation-associated and 35% of *BRCA2* mutation-associated breast cancer has the TNBC phenotype [6, 7]. TNBC may also have HRR deficiency based on other molecular alterations. Recent data suggest approximately 10% of young or familial TNBC patients with no *BRCA1* or *BRCA2* mutation carry inherited deleterious mutations in other breast cancer predisposition genes, particularly in genes involved in HRR such as *PALB2, BARD1, RAD51C, RAD51D,* and *BRIP1* [8-10]. In addition, BRCA1 promoter hypermethylation has been identified in one-third of 377 TNBCs [11]. Deficient HRR leads to activation of alternate DNA repair pathways including the base excision repair and non-homologous end-joining pathways, that require PARP. Increased PARP-1 expression and/or activity in tumor cells have been demonstrated in TNBC [12, 13]. HRR dysfunction sensitizes cells to PARPi that lead to further chromosomal instability, cell cycle arrest, and apoptosis [14, 15]. The PARPi class has shown clinical potential in TNBC [5, 16-18], with a response rate (RR) of 54% in patients with advanced TNBC with germline *BRCA* mutations [18].

Subsets of sporadic TNBC, particularly the basal-like phenotype, share pathologic and molecular features, such as p53 mutation, genomic instability, and sensitivity to DNA cross-linking agents cancers, with germline *BRCA* mutation-associated breast cancers [1]. Platinum agents, such as carboplatin or cisplatin, have clinical activity in TNBC patients with germline *BRCA* mutation [19-21]. Increased *in vivo* activity was reported with the combination of olaparib and cisplatin compared to single agent alone in a *BRCA1*-deficient breast cancer mouse model [22]. We previously reported the combination of olaparib and carboplatin tolerable and active in breast and ovarian cancer patients with germline *BRCA* mutation, in which four of four TNBC patients had either complete or partial responses [5]. However, there are limited data on the activity of PARPi in combination with chemotherapy in patients with metastatic sporadic TNBC. We hypothesized that the addition of olaparib to carboplatin could be safely given and would yield clinical benefit in subsets of sporadic TNBC. Our translational aim was to examine potential biomarkers of response to the PARPi and carboplatin combination.

## RESULTS

### Patients

Patient accrual is shown in the Consort diagram (N = 28; Supplementary Figure 1). Patient characteristics are detailed in Table 1A. Twenty patients had previous negative commercial germline *BRCA* mutational analysis and eight had a BRCAPro score < 10% calculated on the basis of ascertained detailed family history.

**Table 1A:** Patient characteristics (*N* = 28)

Age in years, median	51 (36-75)
ECOG Performance Status, N (%) 0 1	3 (11%) 25 (89%)
Median number of prior regimens^*^	5 (0-12)
Prior chemotherapeutic agents	5 (0-9)
Prior biologic agents	0
Prior hormonal agents	0 (0-4)
Prior platinum exposure	3^**^
Months since last platinum exposure	12 (8-24)
Visceral or non-visceral metastatic disease at screening, N (%) Visceral disease Non-visceral disease	16 (57%) 12 (43%)
Sites of metastatic disease, N (%) Lymph nodes Lung Liver Breast Skin Other (perirectal, cervix, adrenal gland)	19 (68%) 10 (36%) 9 (32%) 3 (11%) 2 (7%) 3 (1 [4%] each)

^*^ All except one patient had been exposed to anthracyclines and/or taxanes-containing regimens, 21/28 (75%) patients were treated with > 3 prior therapy.

^**^ Three patients had previously received platinum-based therapy upon development of recurrent disease; two patients who were not previously platinum sensitive (prior exposure 8 and 12 months prior to study enrollment) developed progressive disease after two cycles of combination olaparib and carboplatin therapy.

### Dose optimization

Patients received olaparib on days 1-7 and carboplatin on day 1 or 2 of a 21-day cycle (Table 1B). One of 6 patients at AUC4 had dose-limiting toxicity (DLT) of grade 4 thrombocytopenia requiring platelet transfusion during the 2-cycle observation period. Increase to carboplatin AUC5 resulted in DLT of grade 4 thrombocytopenia lasting > 7 days in 2 of 2 treated patients, and grade 3 symptomatic hyponatremia with cognitive dysfunction in 1 of 2 patients. The recommended phase 2 dose is olaparib 400mg every 12 hours days 1-7/21 with carboplatin AUC4 day 1.

**Table 1B:** Dose levels and clinical response

Dose Level [DL]	Schedule and Dose		DLT	Best response*** (Duration of response)
	Olaparib oral capsule, bid	Carboplatin IV q 3 wk		
DL 1 (*N*= 4)*	400mg, days 1-7	AUC3, day 1 or 2		1 CR (69+mo), 1 PR (5mo), 1 SD (4mo), 1 PD (1.5mo) *
DL 2 (*N*= 9)**	400mg, days 1-7	AUC4, day 1 or 2	1/6 treated	5 SD (median 3mo), 4 PD (median 1.5mo)
DL 3 (*N*= 2)	400mg, days 1-7	AUC5, day 1 or 2	2/2 treated	1 NE (off due to toxicity), 1 PD (1.5mo)
Expansion cohort (*N*= 13)	400 mg, days 1-7	AUC4, day 1 or 2		4 PR (median 4mo), 6 SD (median 3.5mo), 3 PD (median 1.5mo)

* One patient in DL 1 was replaced because she missed half of the olaparib doses during cycle 1.

**First three patients in DL 2 were replaced due to rapid disease progression including new brain metastases and new chest wall diseases within two cycles. One DLT was observed among six patients on DL 2 and two patients on DL 3.

*** Overall response rate without an exceptional responder: 19% (5 of 27 patients), Disease control rate (CR+PR+SD > 4 months): 4% (12 of 27 patients)

Abbreviations: bid: twice daily, wk: week, mo: months, CR: complete response, PR partial response, SD: stable disease, PD: progressive disease, NE: non-evaluable

### Adverse events

All patients had at least one treatment-emergent adverse event, summarized in Table 2A. Common ( > 10% of patients) non-hematologic events included nausea, fatigue, headache, gastroesophageal reflux, and skin rash. These were mainly mild to moderate in severity, self-limiting, and manageable with standard treatments. Hematologic toxicity was the most common adverse event (Table 2B). Grade 3 and 4 neutropenia was observed in 10 of 28 (36%) but no episodes of febrile neutropenia were observed. 15 of 28 (54%) patients had treatment delays due to hematologic toxicities, and received pegfilgrastim or filgrastim to prevent recurrent delays, starting at cycle 2 (2 patients; non-dose limiting toxicity cohort), cycle 3 (3 patients), cycle 4 (2 patients), cycle 5 (4 patients), cycle 6 (2 patients) and cycle 7 (2 patients). It was continued during all subsequent combination treatment cycles. Grade 3 and 4 anemia was seen in 3 of 28 patients (11%). Six patients (21%) received red blood cell transfusions and 3 (11%) received darbepoetin starting with cycle 3, 4 or 8, respectively. Grade 3 and 4 thrombocytopenia occurred in 3 of 28 patients (11%). Two patients required carboplatin dose reduction due to grade 4 thrombocytopenia lasting more than one week. Four patients (14%) discontinued carboplatin before the planned eight cycles because of myelosuppression. One patient in dose level 3 had both grade 4 thrombocytopenia and grade 3 cognitive dysfunction related to grade 3 hyponatremia; she withdrew from study after one cycle of treatment. MRI was negative for brain metastases, and the electrolyte imbalance and somnolence improved with hydration, pain medication adjustment, and study drug discontinuation. This event was considered possibly related to the study treatment.

**Table 2A:** Drug-related adverse events by maximum grade per patient (*N* = 28)

Adverse Event	Grade 1	Grade 2	Grade 3	Grade 4	Grade 3/4 (%)
**Hematology**					
Lymphocytopenia	10 (36%)	3 (11%)	6 (21%)	0	21%
White Blood Count	8 (29%)	7 (25%)	7 (25%)	1 (4%)	29%
Neutropenia	1 (4%)	7 (25%)	8 (29%)	2 (7%)	36%
Thrombocytopenia	14 (50%)	3 (11%)	0	3 (11%)	11%
Anemia	9 (32%)	10 (36%)	3 (11%)	0	11%
**Gastrointestinal disorders**					
Nausea	11 (39%)	1 (4%)	0	0	0%
Vomiting	3 (11%)	1 (4%)	0	0	0%
Gastroesophageal reflux disease	4 (14%)	0	0	0	0%
Constipation	2 (7%)	0	0	0	0%
Diarrhea	2 (7%)	0	0	0	0%
**Chemistry**					
Hyponatremia	3 (11%)	0	1 (4%)	0	4%
Hypomagnesemia	1 (4%)	0	0	0	0%
Increased AST	4 (14%)	1 (4%)	1 (4%)	0	4%
Increased ALT	4 (14%)	0	0	0	0%
**Other**					
Fatigue	8 (29%)	5 (18%)	0	0	0%
Carboplatin allergic reaction	0	0	0	0	0%
Skin rash	3 (11%)	1 (4%)	0	0	0%
Weight Loss	1 (4%)	0	0	0	0%
Headache	5 (18%)	0	0	0	0%

**Table 2B:** Drug-related hematologic adverse events by dose level (*N*=28)

Adverse Event	Grade 1			Grade 2			Grade 3			Grade 4		
	DL1	DL2	DL3	DL1	DL2	DL3	DL1	DL2	DL3	DL1	DL2	DL3
Lympho-cytopenia	1 (25%)	9 (41%)	0	1 (25%)	2 (9%)	0	1 (25%)	3 (14%)	2 (100%)	0	0	0
Leukopenia	2 (50%)	6 (27%)	0	1 (25%)	6 (27%)	0	1 (25%)	4 (18%)	2 (100%)	0	1 (5%)	0
Neutropenia	1 (25%)	0	0	1 (25%)	6 (27%)	0	0	6 (27%)	2 (100%)	0	2 (9%)	0
Thrombo-cytopenia	3 (75%)	11 (50%)	0	0	2 (9%)	1 (50%)	0	0	0	0	2 (9%)	1 (50%)
Anemia	2 (50%)	7 (32%)	0	1 (25%)	8 (36%)	1 (50%)	0	2 (9%)	1 (50%)	0	0	0

Abbreviations: DL: dose level

DL 1 (4 patients), DL 2 (22 patients) and DL 3 (2 patients)

### Clinical activity

**Figure 1:**
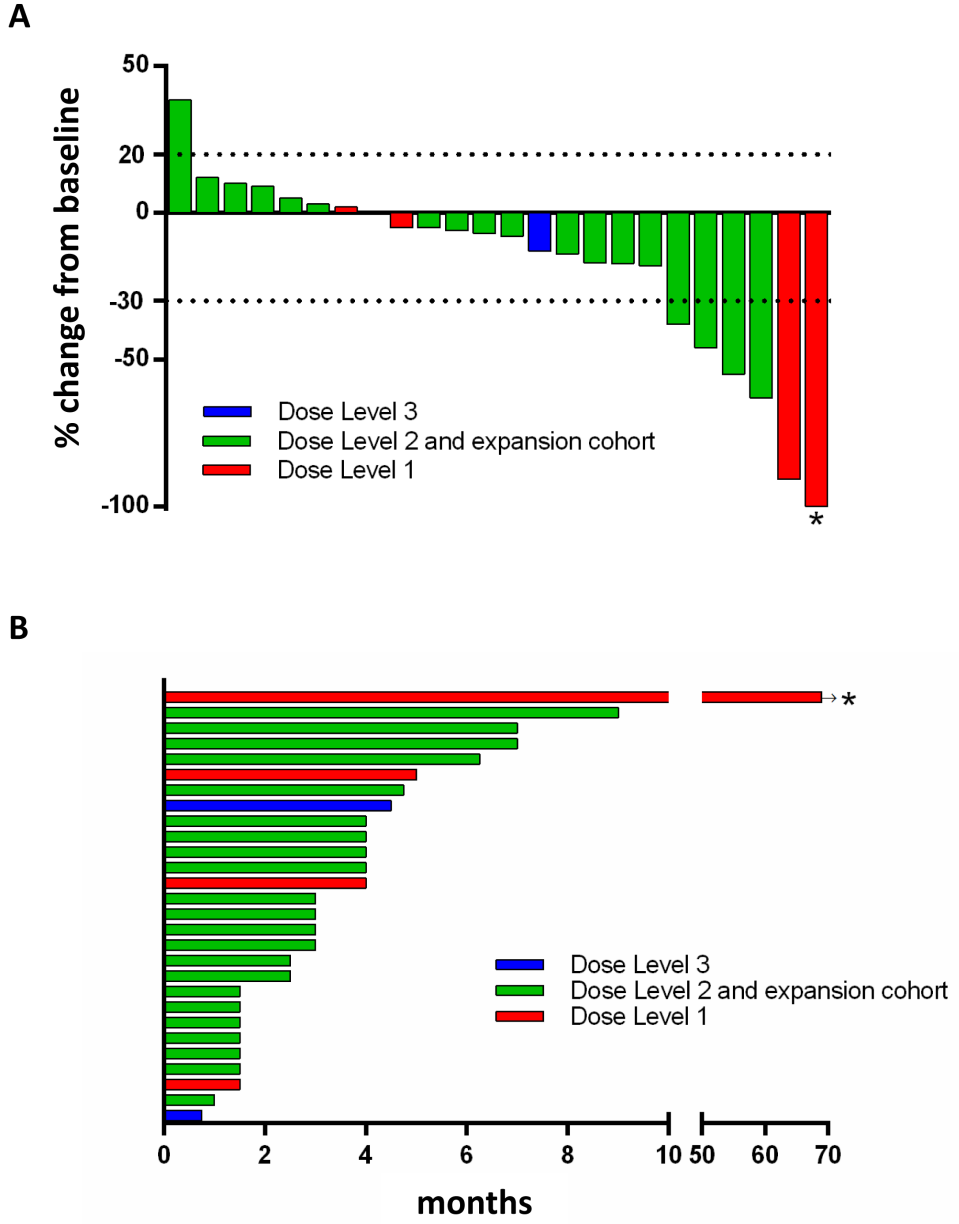
Waterfall plot (A) and duration on the study (B) **A.** Twenty four patients with baseline and subseqent imaging reassessment are shown. Best RECIST response is graphed for each patient. **B.** All 28 patients are shown in a swmmier plot. Four patients became off-treatment due to rapid clinical progression (*n* = 3) and toxicity (*n* = 1) prior to first reassessement scans. Color code defines dose level of treatment with arbitrary patient number assignment. * represents the exceptional responder with ongoing complete response. Dose level 1: olaparib 400mg bid days1-7 with carboplatin AUC3; dose level 2: olaparib 400mg bid days1-7 with carboplatin AUC4; dose level 3: olaparib 400mg bid days1-7 with carboplatin AUC5.

The clinical activity results are summarized in Table 1B and are shown in Figures 1A and 1B. 27 patients were evaluable for response determination. Four patients discontinued treatment during cycle 1 or 2, due to drug toxicity (1, not evaluable for response), disease progression with development of brain metastases (2 patients) or of new chest wall lesions (1 patient). The latter three were counted as disease progression. One patient on the dose escalation cohort achieved a durable complete response (CR; 69^+^ months), 5 had partial response (PR; median 4 months, range 4-7 months) for an objective RR (ORR) of 22% (6/27). Seven patients had stable disease (SD; median 5.5 months, range 4-9 months) yielding a disease control rate of 48% (13/27). Three patients received a carboplatin regimen prior to study entry. The patient with a 24-month carboplatin-free window achieved SD for 9 months. The two patients who had a prior carboplatin regimen, had disease progression at first imaging reassessment.

### Translational studies

#### PAR levels and PARP1/XRCC1 polymorphisms

A significant decrease in the mean value of PAR concentrations was found 24 hours post-olaparib compared with their respective baselines ( 12.68 pg/mL [7.8-35.3 pg/mL] *vs*. 90.43 pg/mL [32.5-172.7 pg/mL], *p* = 0.002). This expected finding demonstates that olaparib achieved pharmacologically effective concentrations. Decrease in PAR incorporation by greater than 50% after one cycle of olaparib/carboplatin was observed in 9 patients and did not correlate with response or PFS.

#### Reverse phase protein array (RPPA)

Protein expression or their post-translational modifications were assessed by RPPA [23]. The relationship between clinical response and pretreatment biopsy lysate expression of 218 proteins or posttranslational modifications of proteins was examined [23]. We explored protein quantity changes between patients who achieved a clinical benefit of ≥ 4 months *vs*. those who did not, and between patients with objective response (CR or PR) *vs*. no objective response (SD or progression). A false discovery rate (FDR) of 5% was used to control for multiple comparisons, yielding four proteins differentially expressed as a function of clinical benefit (Supplementary Table 1A). Additionally, we compared changes in protein quantity between the pre-cycle 2 biopsy and baseline, removing any changing ≤ 10%. No difference was found as a function of objective response. However, significant differences associated with clinical benefit were found for five proteins (FDR = 5%; Supplementary Table 1B). High starting quantities of cyclin D1, collagen VI and YAP-1 were identified in patients who did not derive clinical benefit as defined above. Increases in the three proteins over time were associated with clinical benefit.

#### Apoptosis

The mean apoptotic index (AI) at baseline was 52.1% (39.9- 80.6%) and increased to 60.3% (51.7- 74.6%) post-cycle 1 (*p* = 0.09). The AI fold or % change per patient did not correlate with response or PFS.

#### Exceptional responder BROCA-HR testing

**Figure 2:**
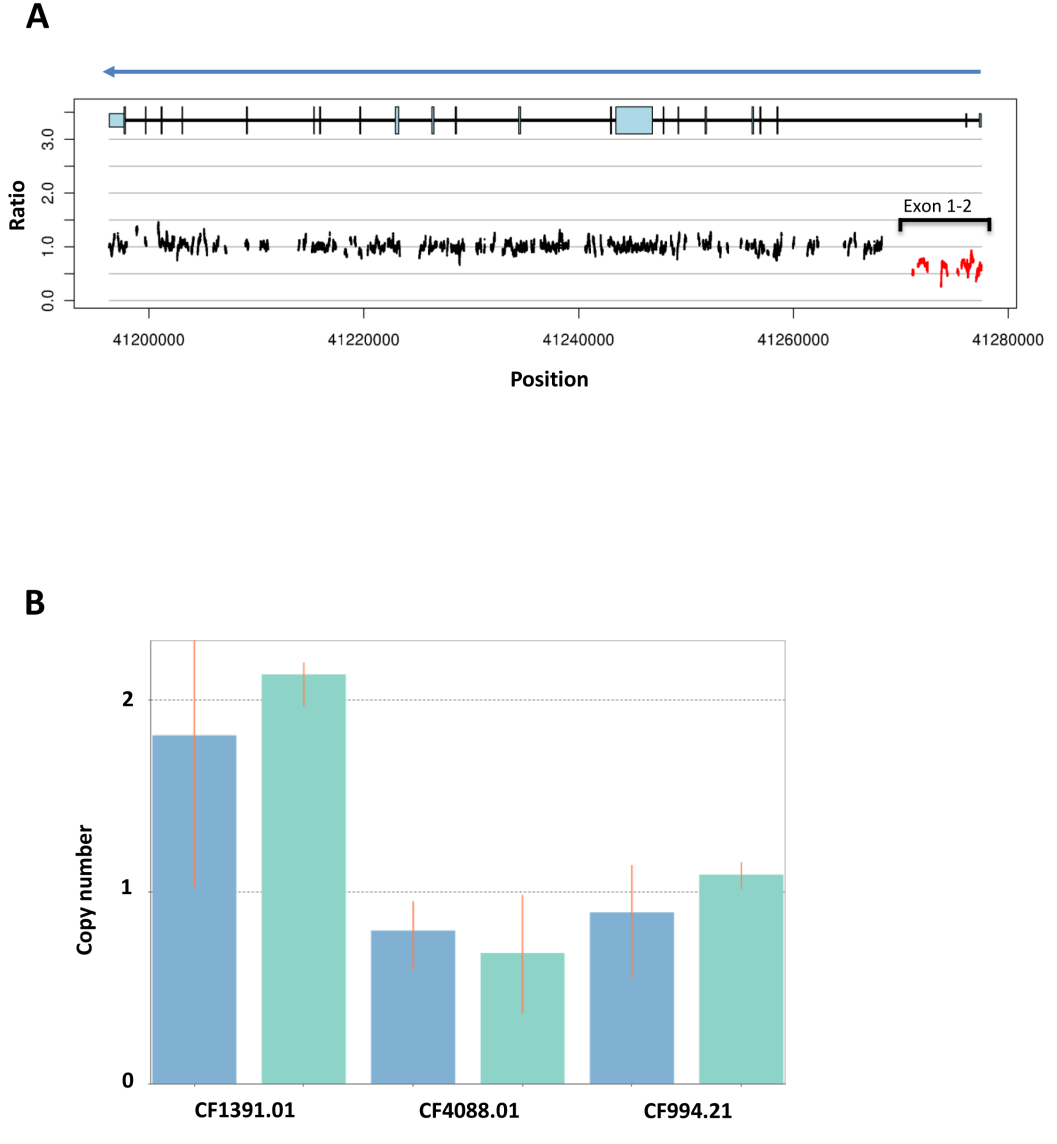
BROCA-HR deep sequencing result of the exceptional responder (A) and validation with qPCR for deletion of *BRCA1* exons 1 and 2 (B) This patient was initially diagnosed with stage I TNBC (T1cNoMo) in 2007 at age 46, treated with lumpectomy, adjuvant radiation followed by 4 cycles of doxorubicin and cyclophosphamide at the local hospital. She was treated for recurrence in 2008 with 2 cycles of docetaxel and capecitabine, followed by 3 cycles of paclitaxel and gemcitabine, after which she underwent surgical excision of a remaining right parasternal mass. This was consolidated with external beam radiation. This was followed by supraclavicular and mediastinal nodal progression in Feb 2010, leading to enrollment in the present study dose escalation cohort. The patient had no known family history of breast, ovarian or prostate cancer. **A.** BROCA-HR readout for *BRCA1* copy number variations (CNVs) based on read depth and split read alignment demonstrates reduced copy number at *BRCA1* exons 1 and 2 for our exceptional responder. **B.** Taqman Copy Number Assay (Applied Biosystems, Carlsbad, CA) with CopyCaller Software v2.0 was used to confirm CNV within DNA obtained from PBMCs of the exceptional responder (CF4088.01). Copy number analysis with two different BRCA1 exon probes; blue columns represent a probe within *BRCA1* exon 1 (*BRCA1* within exon 1 Chr.17:41277232) and green columns represent a probe within an intergenic region between exons 1 and 2 (*BRCA1* intron 1 Chr.17:41276450). CF1391.01 (left), a negative control without *BRCA1* mutation, demonstrates the normal two copies, while the exceptional responder (CF4088.01, middle) and a positive control for a deletion of exons 1 and 2 (CF994.21, right) lack one copy, which is consistent for the two probes.

Germline HRR gene mutational analysis was done for the exceptional responder [24]. She had negative comprehensive commercial *BRCA* testing prior to enrollement on study (Myriad Genetic Laboratories; Salt Lake City, Utah) in 2008. A comprehensive test for gene rearrangements was not performed at that time as she did not meet Myriad-defined criteria [25]. BROCA-HR analysis identified one wild type germline *BRCA1* allele and one *BRCA1* allele containing a deletion of exons 1-2 (Figure 2A), which was confirmed by a TaqMan copy number assay (Figure 2B). This deletion resulted in a stop codon in the mRNA, encoding a truncated BRCA1 protein, defining this as a deleterious germline mutation in *BRCA1*.

## DISCUSSION

Recurrent TNBC is not curable and constitutes a subset of breast cancer with an important unmet therapeutic need. This phase I/Ib study identified olaparib 400mg twice daily in capsule formulation for 7 days, administered with carboplatin AUC 4 every 3 weeks as tolerable with maxium supportive care, and providing modest activity in a group of heavily pretreated TNBC patients. Many patients required growth factor support on progressive cycles, despite this carboplatin dose. Our previously reported cohort of patients with *BRCA* mutation-associated breast or ovarian cancer identified the same olaparib dosage with an AUC5 of carboplatin as the MTD [5]. Prior treatment exposures were similar, although all *BRCA* mutated ovarian cancer patients had prior carboplatin. This difference in the tolerated carboplatin dose between these two groups is unexplained.

The successful addition of PARPi to chemotherapy has been hampered by myelotoxicity, limiting either the dose or exposure duration of PARPi or of the concomitant chemotherapy [16, 17, 26-28]. Our phase I study in women with *BRCA* mutated breast or ovarian cancer found that continuous daily olaparib required more than halving the standard carboplatin dose due to early marrow toxicity [5]. We again found interactive hematologic toxicity in this non-*BRCA* mutation cohort, requiring a lower AUC of carboplatin than recommended in the *BRCA* mutation patients. These results are consistent with a recent phase I study of olaparib and cisplatin for patients with a variety of cancers which found hematologic toxicity requiring intermittent and lower dose olaparib (olaparib 50-100mg capsules days 1-5 or 1-10, with cisplatin dose every 21 days) [16]. A phase I study found that continuous olaparib with weekly paclitaxel was not tolerable in patients with sporadic TNBC, and was halted without identifying a recommended phase II dose [17]. The intermittent dosing of olaparib (200mg capsules on days 1-10) ameliorated some of the toxicity allowing its successful administration with paclitaxel (175 mg/m^2^ every 3 weeks) and carboplatin (AUC4 every 3 weeks) in previously untreated recurrent ovarian cancer patients [26]. The greater olaparib exposure may have been tolerable due to minimal prior treatment exposure and/or to the purported marrow-sparing effects of the addition of paclitaxel [26].

There are limited data on anti-tumor activity of PARPi/chemotherapy combinations in metastatic sporadic TNBC. Gelmon and colleagues found a 2 month PFS using single agent olaparib in unselected patients with TNBC [29]. The olaparib and paclitaxel study of Dent *et al.* treated unselected metastatic TNBC, yielding an ORR of 37% (7/19) in women who received no (15/19) or one prior cytotoxic therapy regimen for metastatic disease [17]. In addition, platinum agents (cisplatin or carboplatin) resulted in 20% ORR (13/66) in metastatic TNBC patients with *BRCA* wild type [30]; a lower ORR (12%) was seen in those who received platinum agents as a second-line therapy [30]. Our patients were heavily pretreated, 75% of them were treated with > 3 prior therapy. Approximately half of our patients had clinical benefit by prolonged stable disease and/or reduction in tumor size by olaparib and carboplatin. Although combination therapy consistently resulted in greater toxicity than olaparib alone, studies suggested some antitumor activity of different PARPi/chemotherapy combinations [16, 17, 31]. Assessment of the value of such combinations requires randomized trial design and may benefit from patient reported outcome and/or quality of life analyses. However, our data suggest that the identification of predictive biomarkers may be necessary before such large scale studies are undertaken.

Exceptional responder analyses provide windows into disease processes. Our exceptional responder had no family history of breast and/or ovarian cancer, and commercial testing identifed no deleterious germline events although large genomic rearrangement testing was not done per Myriad-defined criteria at that time [25, 32]. Large genomic rearrangements constitute approximately 8-14% of *BRCA1* mutations and 1-4% of *BRCA2* mutations [32, 33]. They are more likely to be identified with copy number assays, or whole exome and whole genome approaches, now more commonly applied. Jackson *et al.* reported rearrangements in 42 of 1300 (3.2%) patients referred for *BRCA* mutation testing and only 10 of the 42 had met Myriad criteria [34]. Our patient’s outcome of an over 5-year olaparib-maintained CR identifies her as an exceptional responder, remarkable even for patients with *BRCA* mutation-associated breast cancer. The median duration of response to either olaparib or olaparib/carboplatin is less than one year in *BRCA* mutated patients with locally advanced or metastatic breast cancer [5]. Secondary somatic mutations that restore BRCA protein function are a mechanism of platinum and PARPi resistance in ovarian cancer [35]. The large deletion found in our exceptional responder would not be amenable to such a restorative event, possibly explaining her long response duration. This finding suggests the need to review clinical outcome by mutation type and site.

A challenge remains to identify, develop, and validate effective predictive biomarkers to apply within and beyond this sporadic TNBC patient population [2]. RPPA has been widely employed as a large-scale proteomic analysis for target and biomarker discovery [36-38]. Our proteomic evaluation of paired cases did not confirm our prior findings of altered FOXO3a found in our *BRCA* mutation cohort [5]. New observations identified higher pretreatment expression of cyclin D1, collagen VI, and YAP-1 in nonresponders. Cyclin D1 and collagen VI have previously been described as negative prognostic biomarkers, and their overexpression has been suggested as a possible molecular driver in breast cancer [39-41]. However, in this study, increasing amounts of these proteins across one cycle of therapy was seen in patients who had clinical benefit. YAP-1 is a context-dependent tumor suppressor gene, where loss of expression in breast cancer prognosticates a poor outcome [42] [43-45], thus, potentially supporting the upregulated expression of YAP-1 in responding patients but not readily explaining why high levels prior to treatment would account for the lack of benefit.

Our study has some limitations. Our small sample size may introduce biases in estimating clinical benefit and our translational endpoints were exploratory. Although we controlled for multiple comparisons to reduce the incidence of type 1 errors, all findings will need to be examined and validated prospectively. The RPPA findings were not validated by other methods due to the limited remaining core biopsy samples and should be interpreted with caution. Additionally, we did not assess BRCA1 promoter hypermethylation by immunohistochemistry as part of this study, thus cannot address how many of our patients may have tumors with BRCA1 methylation. Analysis of HRR dysfunction was not prospectively planned, nor were we able to evaluate other patients within this cohort due to insufficient clinical samples, lack of appropriate informed consent with genetic counseling, and some patients have since died. It is possible that additional patients had underlying HRR issues contributing to their treatment response or lack thereof.

There are now a number of PARPi trials in locally advanced, metastatic or recurrent breast cancer. Our findings present an opportunity to further investigate this combination in a subgroup of sporadic TNBC patients, taking into consideration that better results may be observed in women with fewer prior treatments. Although our prospectively planned biomarkers did not correlate with clinical response, our exceptional responder results reconfirm the benefit of carboplatin with olaparib in TNBC with HRR dysfunction due to BRCA1 loss, and supports the consideration for wider spectrum mutational testing or homologous recombination deficiency score testing [46] in women with TNBC.

## PATIENTS AND METHODS

### Patients

This study was approved by the Institutional Review Board of the Center for Cancer Research, National Cancer Institute. Patients with sporadic TNBC (defined by ER < 10%, PR < 10% and no *HER2* gene amplification by FISH or HER2 negative by immunohistochemistry [0 or 1+]) had no identified deleterious germline *BRCA* mutation on prior testing, or a BRCAPro score < 10% [47] calculated on the basis of a detailed family history. Additional eligibility criteria included recurrent, refractory, or locally advanced, unresectable TNBC; measurable or evaluable disease during dose escalation, and biopsiable disease in the expansion cohort; ECOG performance status 0-2; age ≥ 18 years old; normal end organ function except grade 1 anemia, neutropenia, leukopenia, and AST/ALT; no tumor-related therapy for 4 weeks; no platinum therapy for at least 6 prior months irrespective of prior response and no history of NCI Common Terminology Criteria (CTCAE v3.0) grade 4 platinum allergy; no prior PARPi; no infection requiring antibiotics within 7 days; no brain metastases diagnosed or active within the past year. All patients provided written informed consent. ClinicalTrials.gov identifier: NCT01445418.

### Drug administration and determination of MTD

**Figure 3:**
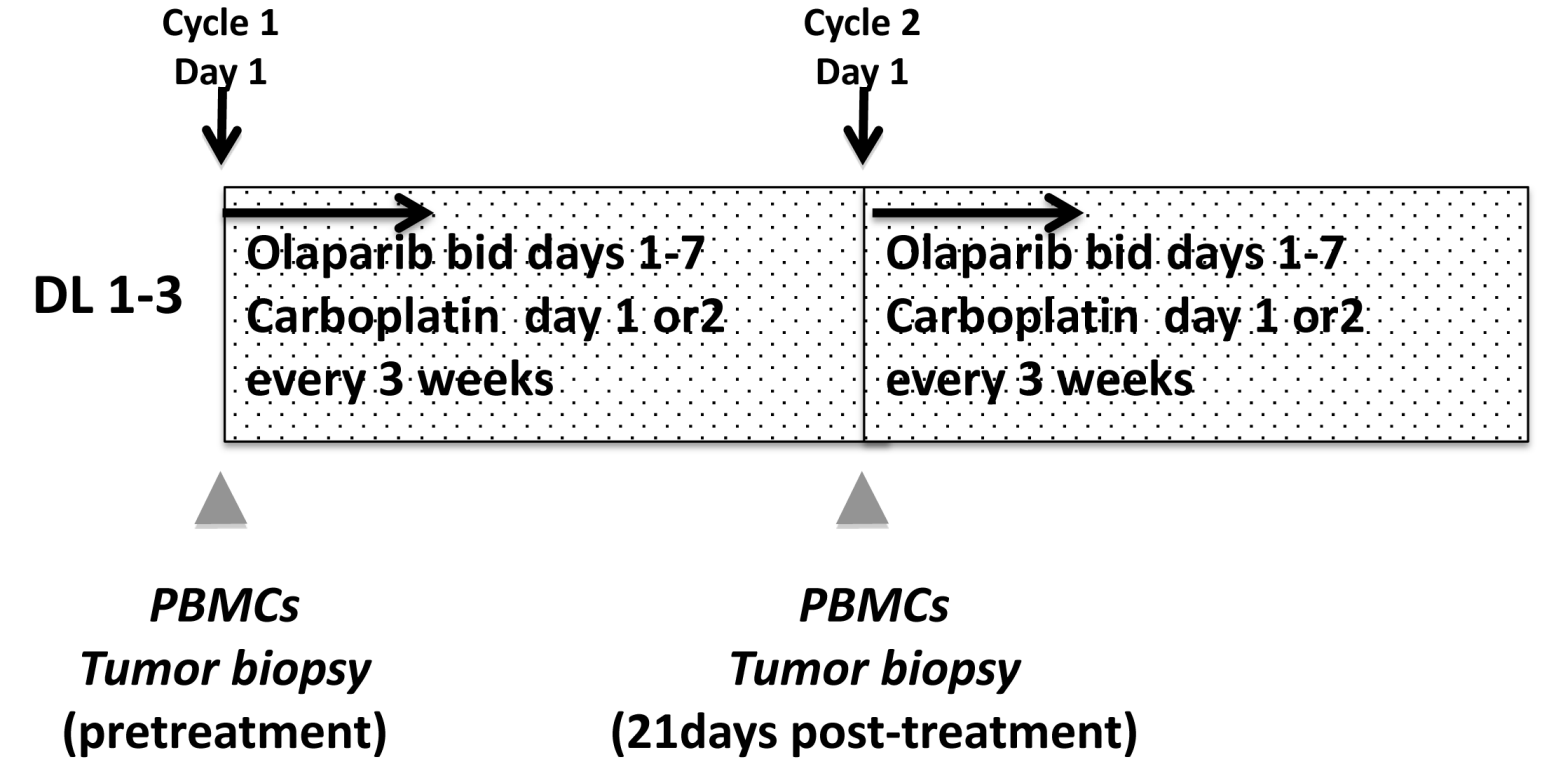
Study schema Abbreviations: DL: dose level, PBMC; peripheral blood mononuclear cells, bid: twice daily

This open label 3+3 dose escalation study examined the combination of olaparib 400mg capsules every 12 hours on days 1-7 with carboplatin AUC3, 4, or 5 on day 1 or 2 (dose levels 1-3), in every 21-day cycles (Figure 3). Carboplatin scheduling was adjusted to accommodate patient travel or when a progressive carboplatin concentration infusion program was required to obviate allergic reactions. No more than 8 cycles of combined therapy was given after which continuous daily olaparib monotherapy at a maintenance dose of 400mg capsules every 12 hours was given. DLTs (CTCAEv3.0) were defined as grade 3 or 4 nonhematologic and grade 4 hematologic adverse events related to study medications occurring during the first two cycles of therapy. The following were exceptions: grade 3 diarrhea, nausea, or vomiting must have been unresponsive to optimal medical management, and asymptomatic grade 3 reductions in electrolytes rapidly reversed with medical management. Grade 3 thrombocytopenia lasting for ≥ 7 days or requiring transfusion, and grade 4 neutropenia for ≥ 7 days or with fever, were dose-limiting. Complete blood counts and serum chemistries were monitored weekly during the DLT period.

Granisetron (days 1-7) and dexamethasone (days 1-4) were given as prophylactic antiemetics during each cycle of the combination therapy and discontinued during olaparib maintenance. Pegfilgrastim was indicated for use if the day 1 absolute neutrophil count [ANC] was less than 1500/mL necessitating a treatment delay, or in subsequent cycles if the day 1 ANC was 1500-1800/mL. It was not allowed during the first 2 cycles of the dose escalation phase. Once initiated, pegfilgrastim was continued during all combination treatment cycles. It was not used during olaparib maintenance therapy. Clinical response was assessed every two cycles by imaging using RECISTv1.0 criteria. Study treatment was discontinued for progression of disease, intercurrent illness, adverse events not recovering to ≤ grade 1 within 3 weeks, and patient preference.

### Translational studies

The translational studies schema is shown in Figure 3. Peripheral blood mononuclear cells (PBMCs) were collected and separated within 4 hours, then stored in aliquots at -80^o^C until use. PBMC DNA poly(ADP-ribose; PAR) incorporation was measured using a commercial PAR immunoassay (Trevigen, Gaithersburg, MD) as previously described [48]. PBMC DNA was isolated and tested for polymorphisms of *PARP1* A762V*, XRCC1* R194W, and *XRCC1* Q399R using a commercial DNA purification kit (Qiagen, Germantown, MD) as reported [5]. Paired tumor biopsies were collected in the expansion cohort patients as described [5]. Percutaneous biopsies were obtained by interventional radiologists under CT or ultrasound guidance using local anesthesia. Samples were processed in real time into optimal cutting temperature compound and stored at −80°C, then cut and stained immediately prior to use [49]. Optimal quality of tissue was defined as paired sequential biopsies with solid tissue areas containing at least 50% tumor cells and less than 25% necrosis [49]. Tissue area was measured and prepared [50] and RPPA was executed by the MD Anderson RPPA Core facility using their 218 antibody protocol including key proteins in DNA repair pathways [23]. Apoptotic cells were counted using the DeadEnd colorimetric TUNEL kit (Promega, Madison, WI) as described [5]. The apoptotic index was defined as the percentage of TUNEL-positive single cells in five high-power fields.

### BROCA-HR mutational analysis

The patient with a 69^+^ month olaparib-maintained CR gave informed consent for germline mutation evaluation of 65 genes [24]. Whole blood DNA underwent massively parallel sequencing with BROCA-HR [51]. Copy number alterations in *BRCA1* exon 1-2 were confirmed by a Taqman Copy Number Assay (Applied Biosystems, Carlsbad, CA) using CopyCaller Software v2.0 (Applied Biosystems).

### Statistical analyses

A protocol-defined expansion cohort was accrued for exploratory biomarker analyses. For any given translational endpoint comparison, 10 paired biopsies were needed to provide 80% power to detect a difference between pre- and on-treatment values equal to one standard deviation of the difference (α_2_ = 0.05). Normalized linear values for RPPA proteins were analyzed against those who had clinical benefit (progression-free survival [PFS] ≥ 4 months) compared to those who did not using multiple paired *t*-tests (GraphPad Prism 6, La Jolla, CA). The Hochberg method was used to control the false discovery rate with Q = 0.05 [52]. Linear correlations between protein levels at initiation of treatment and PFS were examined using JMP 9.0 (SAS Institute Inc., Cary, NCI). The per-patient percent TUNEL change was compared using the Fisher’s exact test (Prism 6). The apoptosis index per patient, and baseline PBMC DNA PAR concentrations were correlated with response and PFS using the Fisher’s exact test. Correlation between polymorphisms in PARP1 and XRCC1 and PFS was tested using the log-rank test, and response using the Fisher’s exact test.

## SUPPLEMENTARY MATERIALS FIGURE AND TABLE


