# Supplementary material for: Phase I/Ib study of olaparib and carboplatin in women with triple negative breast cancer
Source: Oncotarget. 2017 Mar 25;8(45):79175–87. doi: 10.18632/oncotarget.16577 (PMC5668030; doi:10.18632/oncotarget.16577)
Supplement: Supplementary file 1 [file oncotarget-08-79175-s001.pdf]

Phase I/Ib study of olaparib and carboplatin in women with triple negative breast cancer

Supplementary Material

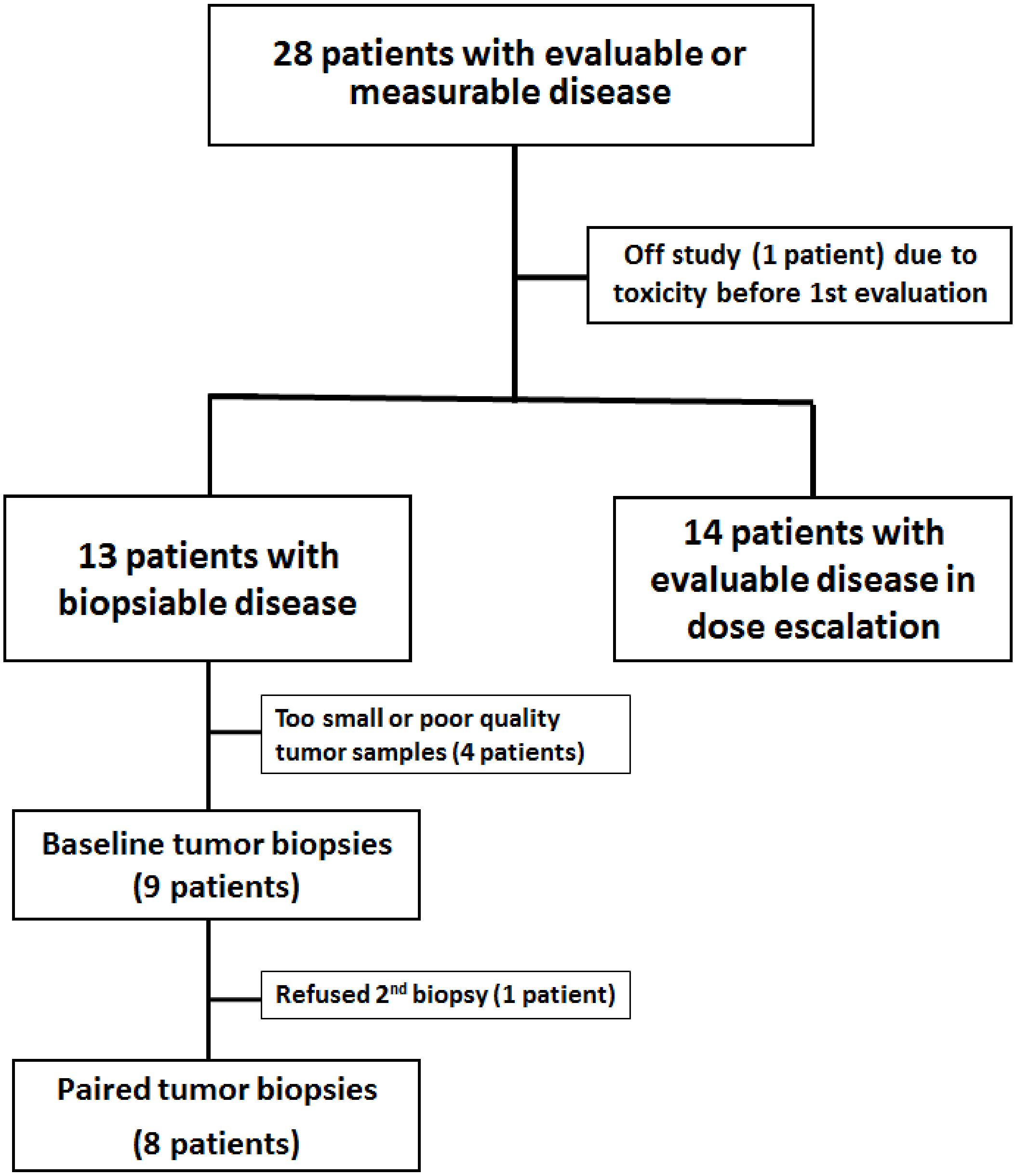

Supplementary Figure1: Consort diagram

Supplementary Table 1

1A. Comparison of Initial Biopsy

	P-value	*Mean (PFS≥4m)*	*Mean (PFS<4m)*	*t ratio*
Cyclin_D1-R-V	0.000000e+000	10.8627	18.4622	22.6259
Collagen VI-R-V	1.901754e-031	5.1149	9.11149	11.8991
Histone H3-R-V	4.585174e-007	3.94642	2.2462	5.06207
YAP-R-E	6.465813e-007	3.10308	4.78084	4.9952

1B. Comparison of Change between pretreatment and post-cycle 1 treatment

	P-value	*Mean (PFS≥4m)*	*Mean (PFS<4m)*	*t ratio*
Cyclin_D1-R-V	0.000000e+000	7.55462	-6.50367	14.0583
Collagen VI-R-V	3.658234e-028	1.85251	-3.57806	5.43057
Myosin-11-R-V	4.428060e-013	3.53862	0.0	3.53862
YAP-R-E	3.968270e-007	0.90701	-1.5611	2.46811
Fibronectin-R-V	0.00044133	1.74507	0.037842	1.70723
